# Hand Compartment Syndrome Due to *N*-acetylcysteine Extravasation

**DOI:** 10.5811/cpcem.2017.9.35152

**Published:** 2017-10-18

**Authors:** Joby Thoppil, Adam Berman, Benjamin Kessler, Payal Sud, Joshua Nogar

**Affiliations:** * Long Island Jewish Medical Center, Northwell Health, New Hyde Park, Department of Emergency Medicine, New Hyde Park, New York; † Staten Island University Hospital, Northwell Health, Department of Emergency Medicine, Staten Island, New York; ‡ North Shore University Hospital, Northwell Health, Department of Emergency Medicine, Manhasset, New York

## Abstract

*N*-acetylcysteine (NAC) is the antidote for acetaminophen (APAP)-induced hepatotoxicity. Both intravenous (IV) and oral (PO) NAC formulations are available with equal efficacy. Adverse events from either preparation are rare. We describe a hand compartment syndrome after extravasation of NAC requiring emergent fasciotomy during phase three of treatment for suspected APAP toxicity. Extravasation injuries leading to compartment syndrome are rare. It is unclear whether IV NAC induced a direct tissue-toxic insult, or functioned as a space-occupying lesion to cause a compartment syndrome. Compartment syndrome from extravasation of NAC is possible. In cases where IV access is difficult, PO NAC is an alternative.

## INTRODUCTION

Acetaminophen (APAP) is one of the most widely used antipyretic and analgesic medications available without a prescription. It is also the leading cause of drug-induced acute liver failure in the United States.^2^–^4^. *N*-acetylcysteine (NAC) is an acetyl derivative of the amino acid cysteine known for its antioxidant properties, and is used worldwide as a well-tolerated and safe antidote for APAP toxicity.^1^ In overdose, APAP depletes endogenous hepatic stores of the anti-oxidant glutathione (GSH), whereas NAC, a GSH precursor, can replete GSH levels.^1^,^4^ NAC either rapidly binds to or detoxifies the highly reactive electrophilic intermediates of APAP metabolism, or it may enhance the reduction of the toxic intermediate of APAP, *N*-acetyl-*p*-benzoquinone imine (NAPQI).^5^ It is most effective in preventing acetaminophen-induced liver injury when given early. It may also be beneficial in reducing the severity of liver injury later in intoxication by several proposed mechanisms, such as improving blood flow and oxygen delivery to the liver, modifying cytokine production and scavenging free radicals.^5^ Therefore, it may also be used empirically when the severity of ingestion is unknown or serum concentrations are not immediately available.^5^

NAC can be administered either orally (PO) or intravenously (IV), with most data demonstrating that they are equally efficacious.^1^–^4^ Some studies go as far as suggesting that PO NAC results in better outcomes than IV NAC by avoiding first-pass hepatic metabolism.^1^,^4^ However, IV NAC is preferentially ordered over the PO form due to practical concerns: 1) PO NAC smells like rotten eggs, which may limit patient adherence; 2) sedation and airway considerations frequently accompany the overdosed patient, thereby rendering PO medications unsafe; 3) the duration of IV NAC therapy is much shorter than the PO dosing scheme (21 hours vs. 72 hours, respectively).^1^–^4^ There are, however, some concerns with the administration of IV NAC, such as rate-related anaphylactoid reactions.^1^,^4^ In this case report, we describe a rare complication of IV NAC.

## CASE REPORT

A 26-year-old male with a psychiatric history significant for polysubstance abuse, undifferentiated psychosis and depression with multiple suicide attempts presented to the emergency department (ED) 12 hours after a suicide attempt. He admitted to an overdose of approximately 55 tablets of aripiprazole (20 mg tabs), diphenhydramine (25 mg tabs), benztropine (1 mg tabs), haloperidol (5 mg tabs) and paroxetine (40 mg tabs). He denied any other ingestions. The patient reported a recent upper respiratory infection. Initial vital signs included a temperature of 98.1° F, pulse of 81 beats per minute, respirations of 18 breaths per minute and a blood pressure of 112/60 mmHg. Upon initial evaluation, the patient was asymptomatic, fully alert and oriented, with an unremarkable physical exam.

The electrocardiogram (ECG) had a normal sinus rate, rhythm and intervals. A 20-gauge IV was placed in the right hand. A complete blood count demonstrated an elevated white blood count (21.6 K/ul, 80.8% neutrophils, 14% lymphocytes). A complete metabolic panel was unremarkable except for a mildly elevated aspartate aminotransferase level of 41 IU/L. Surrogate markers of liver function, such as total protein, bilirubin and PT/INR were also within normal limits. No other metabolic abnormalities were noted. The serum toxicology screen resulted in an APAP level of 15.7 mcg/ml. The bedside toxicology service was consulted due to the elevated APAP level. Despite the patient’s report of ingestion as 12 hours prior to presentation, given the history of multiple suicide attempts and elevated APAP level, IV NAC was administered due to the possibility of a late-presenting acetaminophen overdose. A standard infusion pump was used to control the rate of infusion using standard pressure alarm settings at the appropriate rate for phase of infusion.

During phase three of NAC therapy, the patient began complaining of pain and swelling around the IV site. Exam showed tense swelling of the right hand to mid forearm, pain with passive movement, paresthesias, and a faint but palpable radial pulse. The patient also noted sensation deficits with light touch to the distal fingers as compared to the left hand. Hand surgery was immediately consulted for evaluation of possible compartment syndrome. Compartment pressures were measured as high as 45 mmHg with a delta pressure of 17 mmHg.

The patient underwent an emergent fasciotomy, and the surgical team noted a “rotten egg” odor upon compartment release. The patient was started on broad-spectrum antibiotics due to concerns for infection due to the rotten-egg odor noted during surgery. He was given one dose of vancomycin (1 gram) and piperacillin/tazobactam (3.375 grams). Antibiotics were discontinued once the surgical team was made aware that this odor is characteristic of NAC. No complications were noted post-operatively, and pain, paresthesias, sensory deficits, swelling and range of motion improved over the following two days. The patient continued to improve and was eventually deemed medically stable for transfer to a psychiatric care facility.

## DISCUSSION

Extravasation injury is the inadvertent leakage of a solution into the extravascular space. If the solution leaks into a confined space, it can result in elevated tissue pressures and decreased vascular flow. Specifically, vesicant solutions that extravasate may cause tissue inflammation, ischemia and possible necrosis that may lead to the accumulation of edema fluid in a confined space.^5^–^7^ Acute compartment syndrome develops when the tissue pressure within the fascial sheath surrounding a group of muscles rises to within 30 mmHg of aortic diastolic pressure.^8^ Once compartment pressure reaches this level, microvascular compression results in progressive muscle and nerve ischemia.^8^

**CPC-EM Capsule**

What do we already know about this clinical entity?

There are no documented reports of N-acetylcysteine (NAC)-induced compartment syndrome.

What makes this presentation of disease reportable?

We preferentially use IV formulations of NAC when oral (PO) formulations are just as efficacious. It may be prudent to consider PO formulations when indicated.

What is the major learning point?

NAC is considered a relatively benign medication. However, even benign medications can still cause harm.

How might this improve emergency medicine practice?

Our goal is to have physicians think more carefully about the decision to use intravenous vs. PO formulations when indicated.

In our case, IV NAC likely acted to cause compartment syndrome of the right hand and forearm secondary to extravasation of a large volume of fluid into a confined compartment, as opposed to vesicant injury. This is evidenced by the lack of tissue destruction/inflammation/necrosis in the surgical report after fasciotomy. Despite the use of an infusion pump to control rate, fluid still extravasated into the extravascular space. There were no nursing reports of any malfunction of the pump or any alarms to our knowledge. Furthermore, it is unlikely that the patient could have sabotaged his own infusion, given that he was under constant observation because of the suicide attempt. Progression of care occurred due to symptom presentation and concern for compartment syndrome.

Extravasation injuries resulting in compartment syndromes are rare. However, there have been reports of extravasation injuries complicated by compartment syndromes from contrast dye, chemotherapeutic agents or mannitol. In these instances, it was postulated that the compartment syndromes developed secondary to a combination of excess volume and vesicant tissue injury.^6^–^9^

To our knowledge, no case reports in the literature identify IV NAC as a cause of compartment syndrome. It is still unclear whether the compartment syndrome was due to a volume injury, a vesicant property of NAC, or both. Post-operative reports did not indicate any obvious signs of tissue destruction, suggesting the cause of increased compartmental pressure was a consequence of increased volume in a confined space, as opposed to vesicant injury. In addition, at least one study suggests that the anti-oxidant effect of NAC may prove to be beneficial in limiting injury associated with compartment syndrome.^8^

## CONCLUSION

Monitoring IV sites during antidotal infusions is important to avoid significant extravasation injuries. Although likely very rare, compartment syndrome due to NAC extravasation is a concern with difficult IV access. Oral NAC is a safe, cheap and efficacious alternative to IV NAC in cases where IV access is difficult and no potential contraindications exist. If NAC extravasation does cause compartment syndrome, normal odor characteristics of NAC should be communicated to surgical teams caring for affected patients.
